# Autonomic Nervous System and Stress to Predict Secondary Ischemic Events after Transient Ischemic Attack or Minor Stroke: Possible Implications of Heart Rate Variability

**DOI:** 10.3389/fneur.2018.00090

**Published:** 2018-03-05

**Authors:** Ling Guan, Jean-Paul Collet, Garey Mazowita, Victoria E. Claydon

**Affiliations:** ^1^Department of Medicine, The University of British Columbia, Vancouver, BC, Canada; ^2^Department of Pediatrics, The University of British Columbia, Vancouver, BC, Canada; ^3^BC Children’s Hospital Research Institute, The University of British Columbia, Vancouver, BC, Canada; ^4^Department of Family Practice, The University of British Columbia, Vancouver, BC, Canada; ^5^Department of Family and Community Medicine, Providence Healthcare, Vancouver, BC, Canada; ^6^Department of Biomedical Physiology and Kinesiology, Simon Fraser University, Burnaby, BC, Canada

**Keywords:** autonomic nervous system, stress, heart rate variability, ischemic stroke, transient ischemic attack, prediction

## Abstract

Transient ischemic attack (TIA) and minor stroke have high risks of recurrence and deterioration into severe ischemic strokes. Risk stratification of TIA and minor stroke is essential for early effective treatment. Traditional tools have only moderate predictive value, likely due to their inclusion of the limited number of stroke risk factors. Our review follows Hans Selye’s fundamental work on stress theory and the progressive shift of the autonomic nervous system (ANS) from adaptation to disease when stress becomes chronic. We will first show that traditional risk factors and acute triggers of ischemic stroke are chronic and acute stress factors or “stressors,” respectively. Our first review shows solid evidence of the relationship between chronic stress and stroke occurrence. The stress response is tightly regulated by the ANS whose function can be assessed with heart rate variability (HRV). Our second review demonstrates that stress-related risk factors of ischemic stroke are correlated with ANS dysfunction and impaired HRV. Our conclusions support the idea that HRV parameters may represent the combined effects of all body stressors that are risk factors for ischemic stroke and, thus, may be of important predictive value for the risk of subsequent ischemic events after TIA or minor stroke.

## Introduction

Transient ischemic attack (TIA) and minor ischemic stroke are two types of cerebrovascular ischemic events with mild or transient symptoms and non-disabling consequences (1, 2). However, they are markers of reduced cerebral blood flow and “warning signals” for the possible occurrence of severe ischemic strokes (2). TIA and minor stroke do, therefore, offer a unique opportunity to forestall the onset of permanent brain injury by initiating early treatment (3, 4). Guidelines recommend urgent treatment to TIA and minor stroke, which may reduce the volume of brain damaged by ischemia, promote recanalization of blocked vessels, and decrease the risk of severe ischemic stroke (5, 6). However, these treatments may generate safety concerns related to minor bleedings (7) and/or fatal intracranial hemorrhage (7, 8). Therefore, an urgent and precise risk stratification for TIA or minor stroke is of the utmost importance for medical caregivers to identify high-risk patients and provide personalized treatment. Current risk stratification tools include scoring systems and imaging techniques, all of which have their own individual limitations that inevitably reduce their clinical utility. Accordingly, currently, it remains a challenge to precisely and easily identify the risk of secondary ischemic events after TIA or minor stroke.

People who live with chronic stroke risk factors and suffer an acute TIA or minor stroke episode are at high risk of developing secondary ischemic events (9, 10). Each identified chronic risk factor and acute trigger of ischemic stroke is considered a source of stress for the body. Given the critical role of the autonomic nervous system (ANS) in regulating stress responses (11), it seems possible to determine the comprehensive effect of different stressors by assessing the status of their ANS function. This article is intended to address this issue by reviewing the existing knowledge and evidence and providing an evidence-based deduction on the association between ischemic stroke risk factors, stress, and ANS function in patients with TIA or minor stroke.

We first provide a description of ischemic stroke and TIA with focus on their risk factors and current risk stratification tools. We then present concepts related to stress and the stress response regulatory system, in particular the ANS, whose function can be assessed through the analyses of heart rate variability (HRV). We demonstrate the progressive shift from “stress adaptation” to “stress-related diseases,” with emphasis on the changes to the ANS response throughout this process. Finally, we review evidence in favor of an association between stroke risk factors and ANS dysfunction indexed by impaired HRV parameters.

## Risk Stratification of TIA and Minor Stroke

Beyond all chronic risk factors, TIAs and minor stroke episodes provide additional risk information for secondary ischemic events. Johnston et al. determined that 90 days after emergency diagnosis of TIA, 428 of 1,707 patients (25.1%) developed adverse events including stroke, recurrent TIAs, cardiovascular hospitalization, and death (10). Moreover, Rothwell and Warlow showed that around 17% of ischemic strokes were preceded by a warning TIA; in 43% of cases, this warning TIA occurred within 1 week of the subsequent stroke (12). Although the stroke rate in the first 90 days after an initial event has dropped to 7–13% in recent randomized control trials (13), this overwhelming risk of severe ischemic events after initial TIA or minor stroke underscores the ongoing need for urgent evaluation and treatment of at-risk patients.

The development of secondary ischemic events is generally predicted by assessing the combination of several risk factors (6, 14). AHA/ASA guidelines (6, 14) propose a list of recognized chronic risk factors for ischemic stroke and TIA that are summarized in Table 1. Apart from the traditional chronic risk factors that predispose to the occurrence of ischemic stroke, some acute triggers may precipitate this process (15, 16) (Table 1). Several scoring systems have been widely used in clinical practice to evaluate the risk of early occurrence of severe ischemic stroke after TIA or minor stroke, including ABCD2 (A for age, B for blood pressure, C for clinical feature, D for duration of the symptoms, and another D for diabetes), ABCD3 (the presence of ≥2 TIA symptoms within 7 days added to the ABCD2 score), and ABCD3-I (the presence of abnormal findings on neuroimaging further added to the ABCD3 score). The predictive ability of ABCD2 score is only moderate with an area under the curve (AUC) between 0.55 and 0.7 (17, 18). This may be attributed to the difficulty to qualify the effect of risk factors on an individual basis due to intrapersonal heterogeneity. For instance, “diabetes” has different degrees of severity; and individuals have different ways of coping with the consequences of chronic metabolic stress. Furthermore, other important risk factors, such as smoking, obesity, sedentary life, and psychological stress, as well as other factors not yet identified, are not considered in the classic clinical assessment. Incorporation of the imaging assessment (ABCD3-I) improves the predictive power for future ischemic stroke after TIA or minor stroke, with AUC > 0.8 (19, 20). However, emergency imaging is costly and technology dependent, therefore affecting the universal use with subsequent delays to the scoring process and associated risk assessment. The limitation of current tools for personal risk prediction calls for the development of new valid, precise, and convenient tools to determine the risk of a secondary ischemic event after initial TIA or minor stroke and to direct appropriate medical care for affected patients.

**Table 1:** Identified risk factors for ischemic stroke.

Chronic risk factors		Acute risk factors/triggers
Modifiable	Non-modifiable	
Hypertension Diabetes Dyslipidemia Obesity Atrial fibrillation Cardiovascular diseases Other cardiac events Asymptomatic carotid stenosis Sickle-cell disease Metabolic syndrome Sleep apnea Migraine Hyperhomocysteinemia Hypercoagulability Elevated lipoprotein Postmenopausal hormone therapy Cigarette smoking Heavy alcohol abuse Drug abuse Diet and nutrition Physical inactivity	Age Gender Low birth weight Race/ethnicity Genetic factors	Infections Psychological/mental stress Negative emotions Sudden changes in posture Winter season Diurnal fluctuations Air pollution Surgery Medications Cervical accident and manipulation Pregnancy and postpartum states

One approach that seems promising is to consider that most stroke risk factors listed in Table 1 are also body stressors and therefore affect the ANS response. It may then be possible to determine the overall effect of all life stressors on the body in patients after TIA or minor stroke, by assessing their ANS function. The next section will present evidence of this association, using HRV parameters as markers of ANS function.

## Stress, ANS, and Health

In the central construct of Selye’s stress theory (11, 21), the generalized definition of “stress” describes a state of threatened homeostasis (refers to the stability of physiological systems that maintain life) caused by any form of internal or external disturbing forces, or “stressors.” The person’s life experiences (i.e., the accumulation of stress experiences) contributes to building an idiosyncratic “stress profile” of the individual. This “stress profile,” measured at one specific time, includes both previous and current stress experiences: physical, physiological, psychological, and environmental (11, 22). The “stress response” or “adaptive response” is a counteracting force initiated to neutralize the effects of stressors and re-establish homeostasis. The stress response is a succession of processes that occur in response to the perception of stress by the brain (23, 24). One main stress regulatory system is the ANS, which plays a particularly critical role in modulating the stress response (25, 26).

The ANS is a part of the peripheral nervous system and regulates physiological processes without conscious control. The two major divisions of ANS comprise the sympathetic nervous system (SNS) and the parasympathetic nervous system (PNS). The ANS innervates most organs and controls important physiological and behavioral processes (27). In many physiological situations, the stress response is regulated by the complementary interaction of SNS and PNS. Activation of either SNS or PNS outflow is accompanied by the relative inhibition of the other, suggesting the concept of “sympathovagal balance” (27). The ANS dynamically controls the body response to a range of external and internal stimuli/stressors, providing physiological stability to the body (28). Typically, when the source of stress is acute [i.e., lasting for a period of minutes to hours (22)], the ANS, *via* sympathetic and parasympathetic branches, provides an instantaneous physiological/adaptive response that provokes immediate physiological state alterations through neural innervation of the target organs (24, 28). A typical example of stress response is the physiological inflammatory response (29). The anti-inflammatory response is mostly controlled by PNS, with synergistic input from the SNS (25). As illustrated by Tracey (25, 30), the cholinergic anti-inflammatory pathway represents the afferent branch of the neuronal reflex that modulates local inflammatory responses (25, 30). The efferent branch of the vagus nerve produces acetylcholine that effectively reduces the production of pro-inflammatory cytokines. In addition, both SNS and the humoral anti-inflammatory pathway are triggered, releasing stress hormones that include cortisol and catecholamines to elicit anti-inflammatory effects (25, 30). In this situation, the PNS and SNS act synergistically to control the stress response.

This short-term, tightly controlled regulatory response serves to preserve homeostasis. However, when the source of stress persists for days to months, it is considered to be a chronic stress (22). Chronic stressful conditions represent situations in which environmental demand exceeds the natural regulatory capacity of the body (31). Long-term exposure to these chronic stressors leads to a progressive dysfunctional ANS response to stress, and in particular, to a constrained PNS capacity to control the stress response (21, 32), which may lead to an anticipatory stress response (unpredictable) and a reduced control of the neuroendocrine reaction (uncontrollable) (29, 31). This progressive deterioration of the stress response provides a neuromodulation basis to understand the progression from “stress adaptation” to “stress-related disorders” (32, 33).

**Figure 1:**
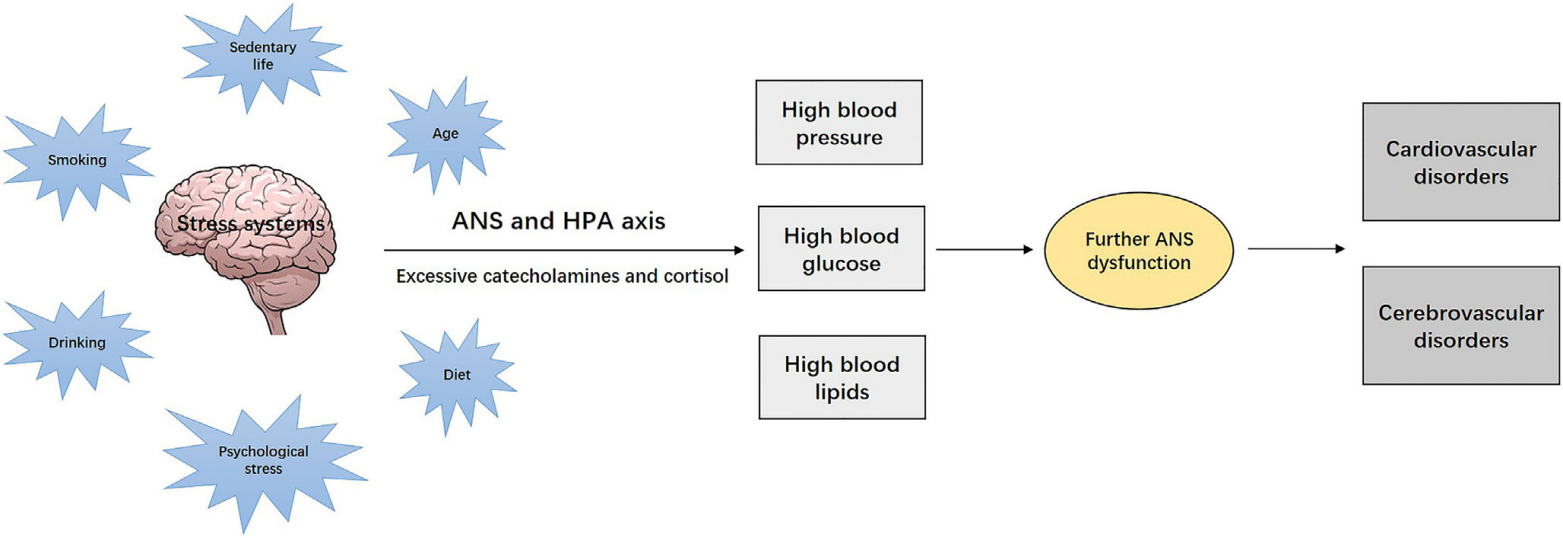
Chronic stress, the nervous system, and development of the stress-related disorders. Chronic stressors, such as aging, diet, cigarette smoking, alcohol consumption, and psychological stress, continuously and cumulatively affect the stress systems [autonomic nervous system (ANS) and hypothalamic–pituitary–adrenocortical (HPA) axis], which lead to excessive production of stress hormones such as catecholamines and cortisol. These stress hormones affect the target tissues and cause various metabolic disorders, such as hypertension, diabetes, and dyslipidemia, which act as “secondary” stressors, and may progressively impair ANS function and ultimately lead to cardiovascular and cerebrovascular diseases.

The established traditional risk factors of ischemic stroke, such as aging, diet, cigarette smoking, excessive alcohol consumption, and psychological stress, are typically chronic stressors that continuously and cumulatively affect the stress systems (ANS and hypothalamic–pituitary–adrenocortical (HPA) axis) (28). The affected neural stress systems will then produce excessive stress hormones such as catecholamines and cortisol, which affect the target tissues and cause various metabolic disorders, such as hypertension, hyperglycemia, and dyslipidemia (28). These metabolic disorders, acting as “secondary” stressors, may further impair the ANS function, creating new pathological cascades, which ultimately leads to cardiovascular and cerebrovascular complications (28). Such disease progression is illustrated in Figure 1. In other words, inappropriate responses to initial stress becomes the source of new stress, leading to a sustained negative cycle of mutual reinforcement toward the development of chronic conditions (28, 33). In this deregulated cascade, it is difficult to distinguish between causes and consequences. The stress system is to a large extent “nonspecific” and meant to interact with internal or external perturbations in a similar manner.

In this chronic stress situation, an additional acute event may extend the overall stress level beyond the range of the physiological and adaptive ANS response. In stroke research, the dynamic nature of stroke development follows such a pattern, in which the acute triggers for ischemic stroke (such as recent infections and TIA episodes) are seen as sources of acute stress to the body that superimpose their effects on the original chronic stress context; and this may increase the overall stress level to a new threshold that precipitates the occurrence of cerebrovascular ischemic events (15, 16, 34).

**Figure 2:**
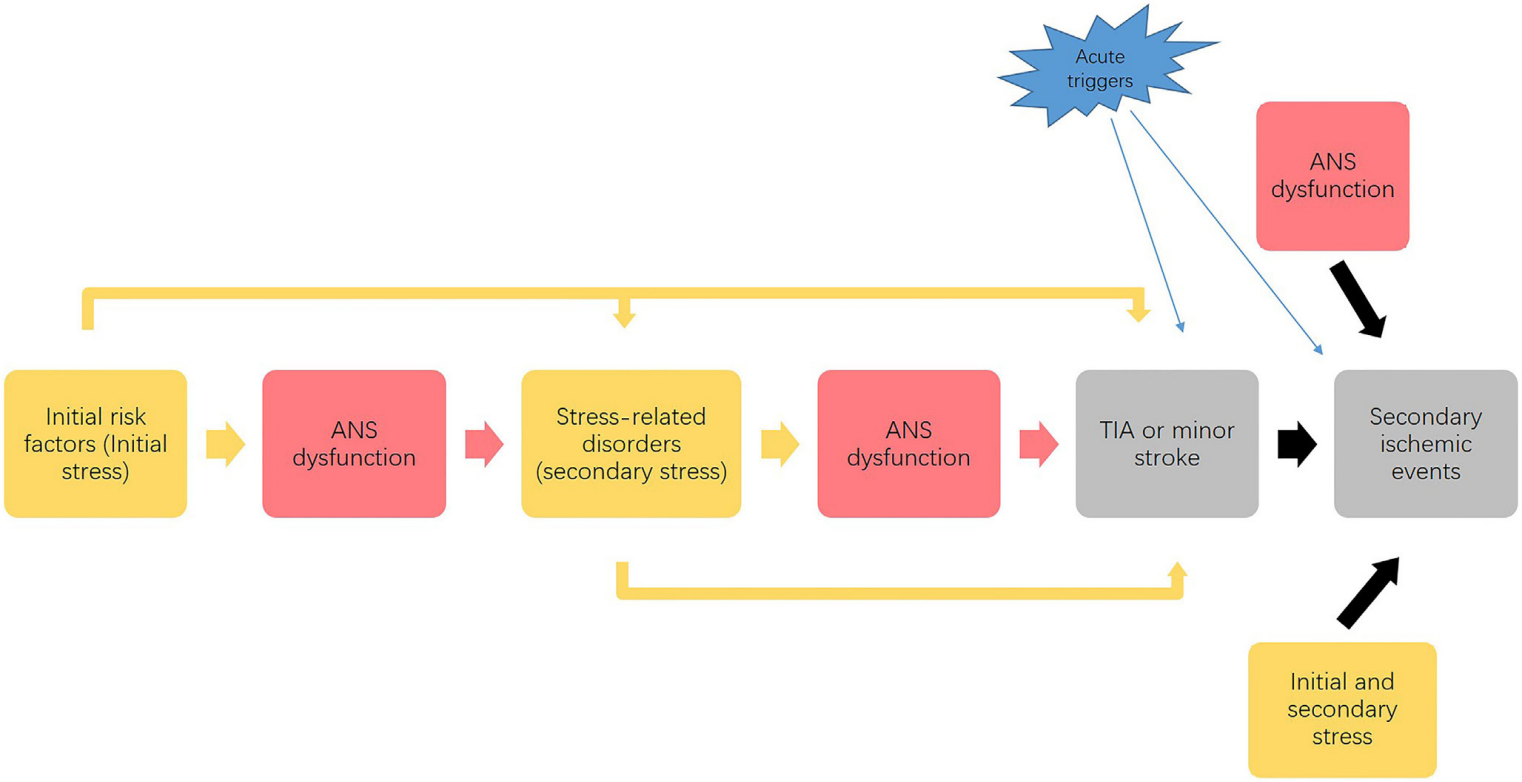
Possible link between stress, autonomic nervous system (ANS) and progression of ischemic stroke. This process illustrates that the initial stress (as risk factors) affects ANS function and causes a dysfunctional ANS response to stress, which combined with the initial stressors causes the development of stress-related disorders. Acting as secondary stressors, these stress-related disorders may further impair ANS function and predispose to transient ischemic attack (TIA) or minor stroke. Finally, the initial and secondary stressors, along with dysfunctional ANS responses, contribute to the development of secondary ischemic events. Acute stressors precipitate the development of both initial TIA and minor stroke events and subsequent ischemic events. This vicious cycle leads to an accumulation of stress that affects the entire body, which potentially promotes the development of initial TIAs and the secondary ischemic events.

This framework supports the association between stress, ANS, and development of ischemic stroke as portrayed in Figure 2. Through the negative spiral described in Figure 2, progressive disease development leads to an accumulation of stress that affects the entire body, with ischemic stroke as the “final endpoint” of the overall effects of multiple stressors.

## ANS Measurement: HRV

The ANS dynamically controls the response of the body to a range of external and internal stimuli/stressors, providing physiological stability in an individual (28). Because most of ANS actions are not accessible to direct and easy physiological testing, in clinical settings the most widely used techniques entail the assessment of an end-organ response to an ANS physiological provocation (35). Some clinical tests for measuring ANS function (35–38) are summarized in Table 2. Among these tests, HRV assessment is a recognized, non-invasive, convenient, and reliable method to measure the ANS function, which will be mainly described in this article. HRV is defined as the fluctuations in the intervals between normal heartbeats (39), and it is mainly based on three analytic methods: frequency domain method, time domain method, and non-linear method (39). The time and frequency domain analyses of HRV are well-developed methods that are recognized as valid and reliable procedures for assessing autonomic function in both clinical and experimental settings (40, 41). Within each analysis, different parameters reflect different aspects of ANS function (Table 3). HRV is generated and analyzed from the ECG waves, which can be recorded from specific devices/software or using 24-h Holter (or other long-term cardiac telemetry) (39).

**Table 2:** Clinical tests of ANS function.

Type of testing	Strength	Limitation
**Testing of cardiovascular modulation** HRV	Non-invasive, convenient, practical, valid, and reliable (described in the following text)	Only application to sinus rhythm – cannot be applied with excessive ectopy or atrial fibrillation

Heart rate and blood pressure assessment at rest or in response to the Valsalva maneuver test, deep breathing, isometric handgrip test, cold pressure test, orthostatic test, head-up tilt test, and baroreflex sensitivity test	Short test duration Assessing both SNS and PNS on cardiovascular modulation	Only assessing ANS response to a rapid change of stress

**Testing of neurotransmitter levels** Catecholamines and acetylcholine assessment	More direct	Invasive Not precise

**Testing of sudomotor function** QSART, thermoregulatory sweat test	Precisely assessing ANS modulation on sweat gland	Not assessing cardiovascular modulation Requiring precautions for electrical safety

**Microneurography** Muscle or skin sympathetic nerve activity	Precisely assessing SNS	Invasive Not assessing PNS Not assessing cardiovascular modulation

*AF, atrial fibrillation; ANS, autonomic nervous system; HRV, heart rate variability; QSART, quantitative sudomotor axon reflex test*.

**Table 3:** Main measures of HRV in frequency and time domains.

	Variable	Definition	ANS modulation and implication
Frequency domain	Total power (ms^2^)	The variance of NN intervals over the temporal segment or 24 h (≤0.4 Hz)	Reflecting overall ANS activity

	ULF (ms^2^)	Power in the ultra low-frequency range (≤0.003 Hz)	Only available in 24-h long-term HRV recording. Representing the influences of many uncontrolled factors

	VLF (ms^2^)	Power in the very low-frequency range (0.003–0.04 Hz)	Representing the influences of the peripheral vasomotor and renin–angiotensin systems, temperature regulation, and other uncontrolled factors

	LF (ms^2^)	Power in the low-frequency range (0.04–0.15 Hz)	Being mediated by a complex mixture of SNS and PNS modulation

	LF norm (n.u.)	LF power in normalized units: LF/(LF + HF) × 100%	Representing the relative value of LF in proportion to the sum of HF and LF and emphasizing the controlled and balanced behavior of the two branches of the ANS

	HF (ms^2^)	Power in the high-frequency range (0.15–0.4 Hz)	Being solely regulated by the PNS, with high HF power representing increased PNS activity

	HF norm (n.u.)	HF power in normalized units: HF/(LF + HF) × 100%	Representing the relative value of HF in proportion to the sum of HF and LF and emphasizing the controlled and balanced behavior of the two branches of the ANS

	LF/HF	Ratio of LF to HF power	Reflecting the balance of SNS and PNS functions

	HF + LF (ms^2^)	Power in the high- and low-frequency ranges (0.04–0.4 Hz)	May represent a more precise indicator of the overall ANS activity. A higher HF + LF value represents increased overall ANS activity, while a lower HF + LF value indicates decreased ANS activity

Time domain	SDNN (ms)	SD of all NN intervals	Corresponding to total power

	SDANN (ms)	SD of the average of NN intervals in all 5-min segments of the entire recording	Corresponding to ULF

	RMSSD (ms)	The square root of the mean of sum of the squares of differences between adjacent NN intervals	Corresponding to HF

	SDNN index (ms)	Mean of the SD of all NN intervals for all 5-min segments of the entire recording	Corresponding to mean of 5-min total power

	SDSD (ms)	SD of difference between adjacent NN intervals	Corresponding to HF

	NN50 count	Number of pairs of adjacent NN intervals differing by more than 50 ms in the entire recording	Corresponding to HF

	pNN50 (%)	NN50 count divided by total number of all NN intervals	Corresponding to HF

*ANS, autonomic nervous system; HRV, heart rate variability; NN, normal – normal interval*.

Due to the dynamic nature of ANS activity, HRV is constantly changing. A single HRV assessment reflects the instantaneous ANS activity at a specific time. According to the uncoupling theory (42), decreased HRV signifies diminished ANS responses; and this process is correlated with disease severity. Conversely, organ recoupling indexed by increased HRV would represent the return of ANS modulation (42). Therefore, assessing the change in HRV between periods can reflect the dynamic/trend of ANS activity over time when stress is changing (40, 43). Accordingly, HRV may serve as a proxy for the neurological mechanisms that guide flexible control of physiology and behavior in the context of stress (44). Through several decades’ progress in HRV research, today HRV measurement is not only an established tool in cardiology research (45) but also increasingly being used in a wide range of clinical and psychophysiological research (46, 47), including informing cardiovascular risk stratification and ischemic stroke prediction (48, 49). Therefore, the critical idea is that HRV may be more than just an index of cardiac function; it may act as an indicator of central modulation of global stress responses. Accordingly, HRV may serve as an easy measurement of the stress regulatory neural network and may provide useful information on the capacity of the body to effectively respond in a stressful situation.

## ANS Dysfunction, Impaired HRV, and Risk Factors of Ischemic Stroke

The relationship between ANS dysfunction and ischemic stroke is complex and bidirectional. ANS dysfunction (a sign of chronic stress) may predict the occurrence of ischemic stroke and, on the other hand, ischemic stroke as a source of new stress affects ANS (50). Ischemic stroke is a source of huge stress characterized by sympathetic predominance, and the associated catecholamine surge may cause cardiac autonomic derangement (51, 52), myocardial damage, and thus possible cardiac dysfunction with increased mortality after ischemic stroke (46, 53). Impaired autonomic function is likely to be a common feature in all ischemic stroke patients (52, 53). Earlier findings showed that: (i) lower HF and/or total power was correlated with a higher risk of incident stroke in adults (49); (ii) other HRV parameters such as dichotomized coefficient of variance of NN intervals (CV%) and power law slope (SLOPE) may also stratify high-risk patients to develop stroke, with 0.68 c-statistic for combined high CV% and high SLOPE (54); (iii) patients with ischemic stroke irrespective of the side of the ischemia in the brain had dysfunctional ANS and decreased HRV [HF, LF and total power (TP)] compared to healthy controls (50, 55); (iv) certain locations of stroke, such as right insular stroke, right middle cerebral artery stroke, and parietal or frontal lobe stroke, were correlated with higher risks of cardiac dysfunction (56), atrial fibrillation (AF) (52, 56), and myocardial injury (57), compared to other sites; (v) acute large strokes, because of the huge stress and catecholamine release, were more likely to cause cardiac dysfunction compared to lacunar strokes; and (vi) poststroke decreased HRV was associated with stroke severity, incidence of early and late complications, and mortality (47, 58). The potential therapeutic effects of parasympathetic activation on ischemic stroke have also been documented (59, 60).

From the perspective of chronic stress leading to disease development (from stress adaptation to stress-related disorders), most risk factors of ischemic stroke can be considered as body stressors (some of them are also consequences of multiple other stressors), with the possible stress cascade as described in previous text. ANS dysfunction assessed by HRV parameters may then reflect the overall effects of different stressors/risk factors (both chronic and acute), including the initial TIA or minor stroke episode. A number of studies (Table 4) report the relationship between autonomic dysfunction measured by impaired HRV and main risk factors of ischemic stroke described in Table 1.

**Table 4:** Summary of main studies assessing the relationship between stroke risk factors and HRV.

Stroke risk factors	Studies	No. of patients	Main HRV measures	Main results	Conclusions
Hypertension	Huikuri et al. (68)	356	HF, LF, VLF, LF/HF, SDNN	– Hypertensives had significantly lower HRV than normotensives: SDNN: 52 ± 19 vs. 59 ± 20 ms, VLF: 103 ± 78 vs. 132 ± 95 ms^2^, and LF: 45 ± 39 vs. 57 ± 43 ms^2^; *p* < 0.01 for all – Normotensives had significant changes in normalized LF and HF (*p* < 0.001) in response to an upright posture, while hypertensives did not	Hypertension results in reduced overall ANS and blunted autonomic responses to a change in body posture

	Liao et al. (65)	2,601	HF, LF, LF/HF, SDNN	– Hypertensives had significantly lower HF, LF, and SDNN than normotensives, *p* < 0.05 for all – People with the lowest quartile of HF had 2.44 (95% CI, 1.15–5.20) fold risk of hypertension than those with the highest quartile of HF	Cardiac autonomic function is associated with hypertension, and reduced vagal function is associated with the risk of developing hypertension

	Singh et al. (67)	2,042	HF, LF, VLF, TP, LF/HF, SDNN	– All HRV measures, except LF/HF, were significantly reduced in hypertensives compared with normotensives, *p* < 0.01 for all – LF was associated with incident hypertension in men (OR, 1.38; 95% CI, 1.04–1.83)	ANS dysregulation is present from the early stage to the established hypertension

Diabetes	Carnethon et al. (71)	8,185	HF, LF, SDNN	Participants with the lowest quartile LF had 1.2 (95% CI, 1.0–1.4, *p* < 0.05) times risk of developing diabetes, compared to those with the highest quartile	ANS dysfunction may be associated with the development of diabetes in healthy adults

	Kudat et al. (73)	62	Most time and frequency domain parameters	– Diabetic patients had lower values in both time and frequency domain parameters than healthy controls, *p* < 0.001. – Diabetic patients with chronic complications had significantly lower values in most HRV parameters than those without complications, *p* < 0.01	Diabetes is a cause of ANS dysfunction, especially in those with microvascular complications

	Tarvainen et al. (72)	472	Most time and frequency domain	– Diabetic patients had significantly lower values in most HRV parameters than healthy controls (*p* < 0.001) – BGL, HbA1c and duration of diabetes were negatively associated with most HRV parameters (*p* < 0.027)	Elevated BGLs cause ANS dysfunction, and this effect is pronounced in long-term T2DM patients

Dyslipidemia	Liao et al. (65)	2,359	HF, LF, SDNN	HF, LF, and SDNN were significantly lower in subjects with one, two, or three multiple metabolic disorders (hypertension, diabetes, dyslipidemia), compared to controls without any metabolic disorder, *p* < 0.05 for all	Metabolic disorders adversely affect cardiac autonomic control

	Christensen et al. (75)	85	SDNN, SDNNi, RMSSD	Plasma total cholesterol and LDL were inversely correlated with all 24-h HRV parameters in both subjects with previous MI or left ventricular dysfunction, and healthy adults	Hypercholesterolemia is associated with ANS dysfunction

	Kimura et al. (77)	175	HF, LF, TP	Triglycerides (124.5 ± 8.6 vs. 97.9 ± 5.9 mg/dl), total cholesterol (224.5 ± 4.3 vs. 210.7 ± 3.6 mg/dl), and LDL cholesterol (127.8 ± 4.6 vs. 115.0 ± 3.5 mg/dl) were significantly higher in low TP group, *p* < 0.05 for all	Reduced overall ANS activity is associated with higher postmenopausal body fat content and blood lipid concentrations

Atherosclerosis	Huikuri et al. (93)	265	HF, LF, VLF, ULF, SDNN, SDANN	The progression of discrete coronary stenosis (change in minimal luminal diameter of negative vessels) was related to all HRV time and frequency domain parameters (*p* < 0.05 for all)	Progression of focal coronary atherosclerosis is correlated with ANS dysfunction

	Manfrini et al. (94)	42	HF, LF, LF/HF	– HF was negatively correlated with plaque burden (assessed by plaque plus media cross-sectional area); while LF/HF was positively correlated with the plaque area – Patients with positive remodeling had significantly lower HF (0.07 ± 0.06 vs. 0.14 ± 0.09 nu, *p* < 0.01) and higher LF/HF (2.1 ± 1.1 vs. 1.4 ± 1.1, *p* < 0.05) than those with negative remodeling	Increasing plaque size and expansive arterial remodeling is associated with vagal dysfunction

Cardiovascular diseases	Kleiger et al. (98)	808	SDNN	RR of mortality was 5.3 times higher in patients with SDNN less than 50 ms than those the with SDNN more than 100 ms	Decreased HRV with increased SNS or decreased PNS may predict cardiac mortality

	Bigger et al. (99)	715	HF, LF, VLF, ULF, TP, LF/HF	ULF and VLF power were strong, and LF and HF power were moderately associated with all cause, cardiac and arrhythmic mortality	HRV could be a good predictor of mortality after MI

	Huikuri et al. (100)	312	HF, LF, VLF, SDNN	Reduced VLF, LF, HF, and SDNN were significantly correlated with higher risks of cardiac arrhythmia events and death 6 weeks after MI, *p* < 0.05 for all	Decreased HRV and ANS dysfunction have prognostic significance after MI

	Jokinen et al. (101)	800	HF, LF, VLF, LF/HF, SDNN	– Low HRV were associated with higher risks of all-cause mortality and cardiac death in univariate analysis – All frequency domain parameters and SDNN improved at 12 months after MI, *p* < 0.05 for all	Changes of HRV parameters have prognostic significance for MI

AF	Perkiömäki et al. (109)	784	HF, LF, VLF, TP	– Patients with AF had significantly lower values of HF, LF, VLF, and TP than those without AF, *p* < 0.05 for all – Hazard ratios for all HRV parameters were significant (*p* < 0.05) in univariate analysis. LF remained significant in the multiple analysis	Patients with AF had ANS dysfunction. Impaired LF may be the best predictor of new-onset AF

	Jons et al. (110)	271	HF, LF, VLF, ULV, SDNN	Reduced LF was correlated with the onset of AF (adjusted HR = 1.6, *p* = 0.034)	Abnormal ANS is independently associated with increased risk of new-onset AF

	Bettoni and Zimmermann (111)	77	Most time and frequency domain parameters	Both HF and LF values increased during the 24 h before the onset of AF; LF/HF progressively increased during the preceding 24 h but had a sharp decrease at 5 min before the onset of PAF	A primary increase in SNS followed by short-term vagal predominance occur prior to the onset of PAF

Aging	Antelmi et al. (124)	653	Most time and frequency domain parameters	All time and frequency domain HRV parameters decreased with age, *p* < 0.001. LF/HF ratio increased from the second to the fifth decade	ANS function declines with increasing age

	Stein et al. (125)	585	HF, LF, LF nu, VLF, ULF, LF/HF	All frequency domain HRV parameters decrease from 65 to 75 (*p* < 0.05) and levels off at age >75	ANS function declines with increasing age, independent of CVD risk factors

Smoking	Harte and Meston (126)	62	HF, LF, HF/HF, SDNN, RMSSD, pNN50	HF, LF, SDNN, RMSSD, and pNN50 were significantly higher among successful quitters compared to unsuccessful quitters, *p* < 0.05 for all	Smoking cessation significantly enhances ANS function

	Yuksel et al. (127)	42	Most time and frequency domain parameters	All HRV parameters were significantly decreased in cigarette, and cigarette and alcohol addicts, compared with controls, *p* < 0.05 for all	SNS activation and PNS inhibition are present in smoking and alcohol addicts

Alcohol consumption	Irwin et al. (130)	28	HF, LF, LF/HF	HF was significantly lower in alcohol-dependent subjects than in controls when awake before sleep and during all sleep stages	Alcohol dependence impairs vagal modulation during sleep

	Thayer et al. (129)	542	RMSSD	RMSSD was significantly lower in high alcohol use group compared to low alcohol use group	Parasympathetic dysfunction is correlated with heavy alcohol use

Sedentary lifestyle	Sloan et al. (131)	149	HF, LF, SDNN	– Aerobic activity led to a significant increase in HF (lnHF = 0.25, 95% CI = 0.09–0.41, *p* < 0.05) compared to baseline – Men had increased SDNN (lnSDNN = 0.12, 95% CI = 0.04–0.20, *p* < 0.05) after aerobic activity compared to baseline	Aerobic activity enhances ANS function

	Earnest et al. (132)	365	HF, LF, VLF, TP, SDNN, rMSSD	Both HF and rMSSD improved significantly in the 8 and 12 weeks exercise for all age groups (*p* < 0.05 for all)	Long-term exercise improves PNS activity

Psychological stress	Hall et al. (138)	59	HF, LF/HF	HF was significantly lower in the stress group than in controls during the entire sleep period (*p* < 0.02). LF/HF was higher in the stress group during NREM sleep (*p* < 0.05)	Acute stress was associated with decreases in parasympathetic modulation during entire sleep periods and increases in sympathovagal balance during NREM sleep

	Miu et al. (137)	63	HF, LF, LF/HF	HF was significantly different between subjects with high and low trait anxiety (33.15 ± 9.45 vs. 38.31 ± 10.76 ms^2^), and between stress and relaxation (31.81 ± 12.6 vs. 37.93 ± 15.21 ms^2^), *p* < 0.05 for both	Psychological stress is associated with autonomic dysfunction

Infections	Toweill et al. (140)	30	HF, LF, LF/HF	– HF and LF were significantly lower in patients with septic shock compared to those with sepsis (LF: 2.68 ± 0.24 vs. 3.37 ± 0.17 bpm^2^; *p* < 0.03 and HF: 2.18 ± 0.14 vs. 2.79 ± 0.23 bpm^2^; *p* < 0.04) – HF and LF were improved during recovery phase, *p* < 0.001 for both	The degree of autonomic dysfunction may help differentiate sepsis, septic shock, and recovery states

	Schmidt et al. (142)	236	HF, LF, VLF, TP, LF/HF, RMSSD, SDNNi	Changes in HRV (VLF, TP) after subarachnoid hemorrhage reflect both infectious and delayed ischemic events and complications	HRV may have prognostic values on infection and ischemic events after subarachnoid hemorrhage

*AF, atrial fibrillation; ANS, autonomic nervous system; BGL, blood glucose level; HRV, heart rate variability; LDL, low-density lipoprotein; PAF, paroxysmal atrial fibrillation; T2DM, type 2 diabetes; HbA1c, glycated hemoglobin; HF, high frequency; LF, low frequency; VLF, very low frequency; TP, total power*.

### Metabolic Disorders and ANS Dysfunction

Metabolic disorders including hypertension, hyperglycemia, and dyslipidemia are sources of chronic stress to the body and well-documented modifiable risk factors for both first and recurrent ischemic stroke (6).

It has been confirmed for several decades that SNS hyperactivity and PNS underactivity are central components in the etiology of early and borderline hypertension, as well as sustained essential hypertension (61, 62). A “neuro-adrenergic” overdrive (i.e., hyperactivity of the SNS) was found in both hypertensive males and females, in young and elderly people with hypertension (63). Therefore, beta-blockers that are competitive antagonists of the beta-adrenergic receptor are widely used to control hypertension (64). Numerous early studies have demonstrated the association between hypertension and autonomic dysfunction measured by lower values of both time (SDNN, SDANN, and RMSSD) and frequency (HF, LF, VLF, and TP) domain HRV parameters (65–68).

Similarly, abundant evidence has demonstrated that an altered balance of PNS and SNS, mainly explained by attenuated parasympathetic activity and a relative elevated sympathetic activity, are causative factors that trigger a cascade of inflammatory/stress responses in the development and progression of diabetes (69, 70). The effect of stress and catecholamines on impairing glycemic control supports the involvement of SNS in the pathophysiology of diabetes (71, 72). A number of studies have shown that an attenuated PNS activity contributes to the development of insulin resistance and diabetes with significantly reduced values of all HRV parameters (HF, LF, TP, SDNN, RMSSN, and pNN50) in diabetic patients, compared to healthy controls (71–73).

Finally, dyslipidemia has also been shown to correlate with SNS activation and PNS suppression (74). High levels of low-density lipoprotein (LDL) and total cholesterol are associated with low HRV values (HF, LF, TP, SDNN, and RMSSD), suggesting an impaired ANS function in individuals with dyslipidemia (75–77).

### Arterial Stiffness, Atherosclerosis, and ANS Dysfunction

Arterial stiffness is associated with a degenerative process affecting mainly the extracellular matrix of elastic arteries with aging and other risk factors, such as high blood pressure (78, 79). On the other hand, arterial stiffening may result in changes to vessel walls and activate a number of complex mechanisms involved in the process of atherosclerosis with associated development of cardiovascular events (80–82). Both arterial stiffness and atherosclerosis are sources of chronic stress to the body and independent risk factors for ischemic stroke (83–85). Atherosclerosis is responsible for the thrombosis and occlusion of large brain arteries (large-artery atherosclerosis subtype), associated with an increased risk of small-vessel stroke (lacunar subtype), and partially contributing to embolism (cardioembolic subtype) (83).

Arterial stiffness is associated with sympathovagal imbalance, particularly increased sympathetic activity (86, 87). In a normal state, the ANS and the endothelium work together to maintain the vascular tone. There is a tonic balance between the release of vasodilating factors from the endothelium and vasoconstricting factors from sympathetic nerve terminals (88). This balance acts on the vascular smooth muscle cells to maintain the appropriate vessel tone (89). Impaired ANS regulation contributes to abnormal changes in endothelial cells, resulting in endothelial dysfunction. Some mechanisms may be that the high SNS activity and increased catecholamines influence the inflammatory process, increase the uptake of LDLs, activate beta- and/or alpha-adrenergic receptors, and finally cause endothelial damage (88). Reduced total power and HF values, as well as higher LF/HF ratio, have been shown to be correlated with reduce arterial distensibility in patients with hypertension (90). Moreover, exercise with dietary restriction improves cardiac autonomic activity reflected by increased SDNN, RMSSD, TP LF, and HF and decreased LF/HF; and this enhanced cardiac autonomic modulation (assessed with decreased LF/HF) was associated with decreased arterial stiffness (91).

According to the prevailing theory proposed by Ross (92), atherosclerosis development is predominantly a cascade of inflammation/stress response-mediated events, from initiation through progression, rupture, and ultimately to the thrombotic and embolic complications. During the process, ANS dysfunction is characterized by the stimulation of SNS and downregulation of PNS, with subsequent impairment of the tight control of inflammatory responses (25). Decreased parasympathetic function (decreased HF, normalized HF, and increased LF/HF) and increased sympathetic function have been reported to correlate with the progression of coronary artery atherosclerosis (93) and coronary artery remodeling (94). In addition, the ANS also plays a crucial role in thrombogenesis (95).

### Cardiovascular Diseases and ANS Dysfunction

The contribution of autonomic dysfunction to the development of cardiovascular diseases has been well illustrated (32, 48). Increased SNS promotes vasoconstriction, increases platelet aggregation and pulse and blood pressure, and decreases fibrinolysis, while decreased PNS leads to reduced arterial pressure and cardiac output. These pathophysiological changes increase the risk of thrombosis due to sluggish flow and arterial wall collapse, and the risk of consequent cardiovascular disorders (96, 97). From the 1980s to recent times, numerous studies have clearly demonstrated that reductions in both time and frequency domain parameters of HRV (SDNN, ULF, VLF, LF, and HF) were present in MI survivors and were correlated with poor prognosis (such as mortality and arrhythmia events) after acute MI (98–100). These HRV indexes are depressed at the early phase of acute MI with substantial improvement during recovery (101, 102).

### Atrial Fibrillation (AF) and ANS Dysfunction

Atrial fibrillation is one of the high-risk cardiac sources for cardioembolic ischemic stroke (14, 103). All types of AF, including paroxysmal, persistent, and permanent, are associated with around fivefold increased risk of ischemic stroke (104, 105). Histological studies have shown that the pulmonary veins where the AF impulses originate are richly innervated by both sympathetic and parasympathetic nerves (106). As early as 1978, Coumel et al. reported that cardiac autonomic dysfunction might predispose patients to develop paroxysmal atrial fibrillation (PAF) (107). Later studies on HRV and AF have further determined the crucial role of the ANS, with relative increased SNS and decreased PNS, contributing to the development, progression, and maintenance of AF (108). Patients with AF are reported to have significantly lower values of HF, LF, VLF, and TP and increased LF/HF, compared to those without AF (109, 110). Interestingly, another study showed that LF/HF increased during the preceding 24 h but decreased sharply at 5 min before the onset of PAF, which may suggest a primary increase in SNS followed by short-term PNS predominance prior to the onset of PAF (111). In addition, the classic treatment for all types of AF is the administration of beta-blockers to inhibit the SNS (112, 113).

### Cardiac Surgery and ANS Dysfunction

Cerebrovascular complications including ischemic stroke and TIA are common after cardiac surgery, especially heart transplantation (114). The incidence of stroke increases with the number of preoperative stroke risk factors, such as a history of hypertension, diabetes, smoking, stroke, and vascular diseases (115). Heart transplantation interrupts the parasympathetic vagal neurons and the intrinsic postganglionic sympathetic nerve fibers traveling from the stellate ganglia to the myocardium, which may cause axonal Wallerian degeneration and thus cardiac denervation (116). The cardiac denervation will cause the lack of parasympathetic connections and abnormal cardiopulmonary baroreflexes, which alters autonomic regulation on cardiovascular function, resulting in lower HRV, cardiac index, abnormal catecholamine levels, and higher heart rate (117–119). Moreover, cardiac surgical procedures often cause AF. As described in the last section, this is associated with an increased risk of embolic strokes, and the use of beta-blockers aims at preventing such strokes (120, 121).

### Aging and ANS Dysfunction

Aging, as a marker of stress experience, can be seen as a chronic body stressor, which leads to ANS alteration (122, 123). Autonomic dysfunction in seniors is also attributed to several main features associated with aging, such as loss of neurons, loss of axon branches, alterations in neurotransmitters, and degenerative changes in effector organs innervated by autonomic nerves (122, 123). Many clinical symptoms associated with aging, such as increased blood pressure and decreased baroreflex function, are associated with relatively elevated SNS and diminished PNS activities that elicit inadequate autonomic responses to physiological stressors (122, 123). Previous studies have shown that elderly people have significantly lower values of HF, LF, and TP powers than young people (124, 125).

### Unhealthy Lifestyle and ANS Dysfunction

Unhealthy lifestyles including cigarette smoking, heavy alcohol use, sedentary lifestyle, and others are all correlated with autonomic dysfunction. Lower HRV values (HF, normalized HF power, LF, TP, and SDNN) and higher LF/HF ratio are found in smokers than in non-smokers in both early and recent studies (126–128). Similarly, people suffering from heavy alcohol abuse had decreased RMSSD and HF and increased LF/HF ratio, compared to controls (129, 130), indicating an impaired vagal function in alcoholics. Further, sedentary lifestyle is also related to autonomic imbalance, primarily suppressed PNS activity (decreases in HF, pNN50, and RMSSD), while exercise may improve autonomic function with increases in HRV parameters (131, 132).

### Psychological Stress and ANS Dysfunction

Psychological stress, caused by occupational, familial, or life events, is recognized as a potential contributor to an individual’s perceptions of stress (133) and possibly interacts with the ability to cope with specific stressors (134). Various components of psychological stress, including self-perceived stress, stressful life events, and poor coping ability, are associated with an increased risk of ischemic stroke (135). High levels of chronic psychological stress lead to continuous activation of the stress system, with prolonged secretion of stress mediators such as catecholamines and cortisol (24, 133), which eventually promotes SNS activity and suppresses PNS activity (28). Studies have shown decreased HF and normalized HF as well as increased LF/HF in perceived psychological stressful situations (136–138). These findings indicate a lower cardiac vagal activity in people who perceive a higher level of psychological stress.

### Recent Infections and ANS Dysfunction

Infection possibly contributes to atherosclerotic plaque pathology *via* inflammation, by activating inflammatory cytokines that accelerate the maturation of plaques and promote plaque instability and rupture (139). Previous studies have shown that patients with infections have decreased HF, LF, and TP, as well as increased LF/HF ratio, compared to both their recovery states (140), and healthy controls (141). Changes to markers of HRV have also been identified in association with the stage and deterioration of infection, which supports the use of HRV as an indicator of illness severity (140, 141). In addition, a recent study shows that changes in HRV parameters could predict the onset of infection and ischemic events after subarachnoid hemorrhage (142).

## HRV-Based Comprehensive Stress Model

According to the stress theory, and association between stroke risk factors, stress, and ANS/HRV, it is possible to establish a “comprehensive stress model” using HRV as a marker of ANS activity and adaptation to stress. This model would represent the overall effects of stroke risk factors and could be used to identify personalized risk of experiencing a secondary ischemic event after TIA and minor stroke.

**Figure 3:**
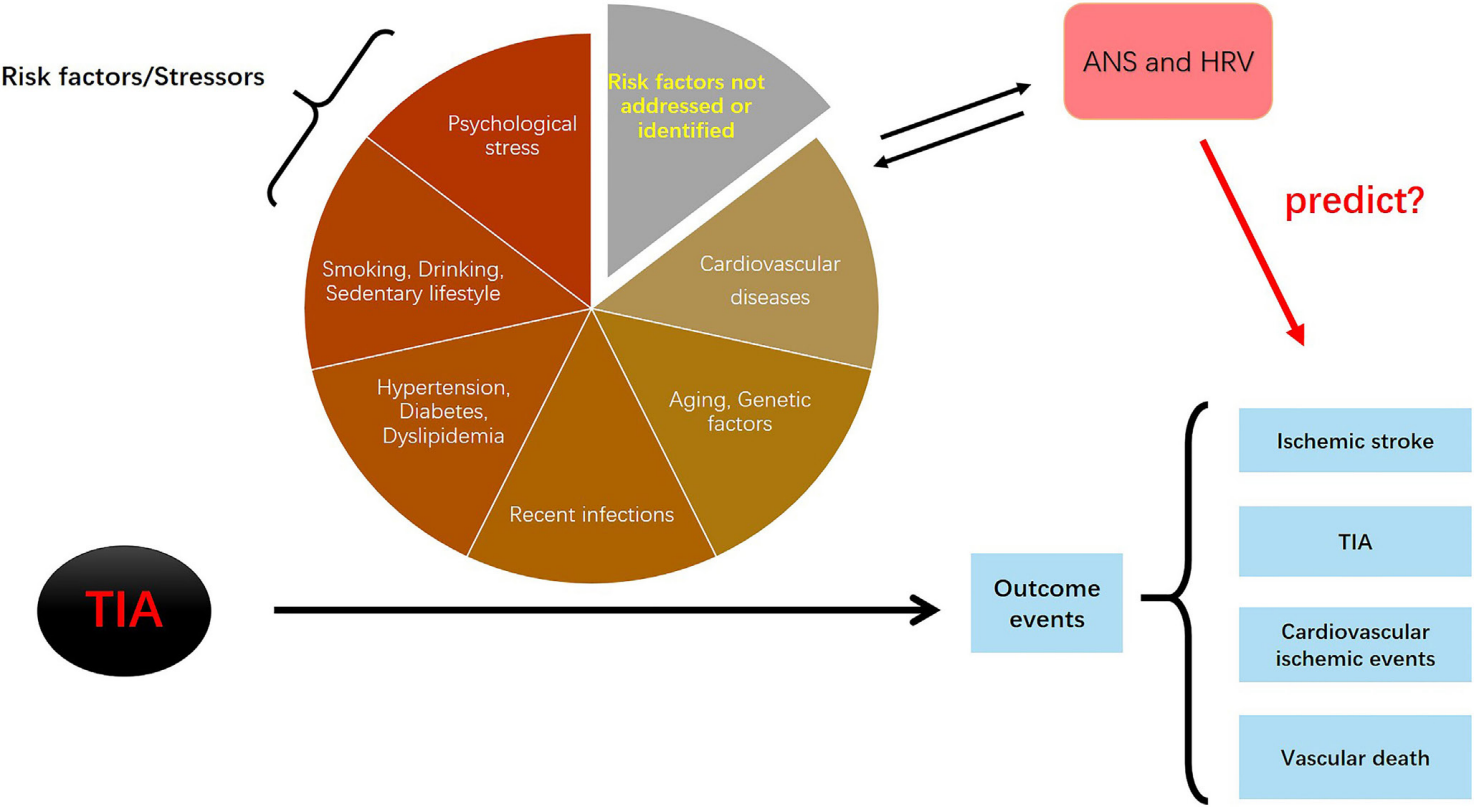
The logic of the heart rate variability (HRV)-based stress predictive model. All risk factors (stressors) have effects during the progression from transient ischemic attack (TIA) to the development of outcome events (ischemic stroke, TIAs, cardiovascular diseases, and vascular death). Autonomic nervous system (ANS) is directly affected by the risk factors/stressors; on the other hand, dysfunctional ANS activity conversely contributes to the development of risk factors/stressors. If correct, this model would suggest that whether HRV parameters (as markers of ANS) can predict the occurrence of secondary outcome events.

The HRV-based comprehensive stress model refers to a model that comprises HRV parameters and multiple dimensions of stress variables, including chronic and acute, physiological, and psychological. The assessment of HRV measures the objective physiological response to stress factors. For an individual, HRV values may represent the comprehensive effect of “multiple stressors” at a given point in time. The HRV-based theoretical model is presented in Figure 3.

### Identification and Selection of HRV Predictors in the HRV-Based Model

Early studies have shown the 24-h rhythm of HRV in both healthy individuals and those with disease (for example, diabetes, chronic stable angina, or coronary artery disease) (143–145). In general, the absolute values of all HRV parameters (HF, LF, VLF, and TP) in healthy individuals are consistently higher than those in diseased people during the entire 24 h (143, 145). In normal conditions, ANS activity has a circadian rhythm with PNS increasing during nighttime and SNS activating during daytime. This circadian rhythm leads to a 24-h HRV rhythm. However, in patients with chronic disease, HRV parameters fail to show normal diurnal changes (143, 145). Among these HRV parameters, HF provides the highest discriminative ability between patients with chronic disease and healthy individuals; more specifically, people with diabetes, chronic stable angina, or coronary artery disease show lower HF values, less day–night rhythm and less daytime rhythm in HF, compared to healthy individuals (144, 145).

Based on the expected values and changes in HRV during a 24-h period, two-dimensional HRV measurement, including HRV absolute values at a given time (as indicators of static ANS activity) and HRV changes over time (as indicators of dynamic ANS activity), can be considered for inclusion in HRV-based predictive models. Several further hypotheses regarding the identification and selection of HRV predictors are made to establish the HRV-based comprehensive stress model to predict secondary ischemic events after TIA or minor stroke.

• *Regarding types of HRV parameters*: HF may be the primary HRV parameter to select because it is a precise indicator of PNS activity and should be lower in patients under stress. Normalized HF that represents the balanced PNS and the proportion of PNS to ANS may also be selected. TP as a marker of overall ANS activity may be included in the assessment. HF + LF, although not a traditional parameter, may be considered as the fraction of HRV that can be totally explained by ANS modulation based on the physiology of HF and LF and thus may represent a more precise indicator of the overall ANS activity. Absolute values of HRV parameters are indicators of people’s health condition; therefore, a lower HRV value may be associated with worse health conditions and a higher level of stress, and thus, a higher risk of developing secondary ischemic events after TIA or minor stroke.
• *Regarding time periods*: In healthy situations, HF power that presents the PNS activity is increased during the night and relaxation period, such as napping. The morning time, especially the few hours after waking up is the period of SNS activation, which leads to the decrease of both absolute and normalized values of HF (6:00 a.m.–9:00 a.m.). HF recovers after 9:00 a.m. and remains comparatively stable with small fluctuations during daytime and early evening (around 9:00 a.m.–9:00 p.m.) (143, 145). Therefore, 9:00 a.m.–12:00 p.m. may be used to represent “morning time” to avoid the sharp decrease in PNS and increase in SNS immediately after waking up (144). 3:00 p.m.–6:00 p.m. may represent afternoon time to avoid the effect of the midday napping. 12:00 a.m. to 3:00 a.m. is to represent the night time because it is most likely period that people are sleeping.
• *Use of HRV changes between day and night*: HRV demonstrates greater day vs. night discrepancies among healthy populations than among people with diseases. It is therefore reasonable to postulate that decreased amplitude of HRV changes between day and night may suggest less restoration of ANS activity and less control of stress and thus a higher risk of secondary ischemic events.
• *Use of HRV changes during daytime*: If people are under stable conditions, HRV remains stable during daytime, i.e., from late morning (after 9:00 a.m.) to afternoon (around 6:00 p.m.) (144, 145), which support the uncoupling and recoupling theories explained in previous text. Accordingly, decreases in HRV parameters during daytime may indicate less rebound capacity of the body or deterioration of health condition (excessive stress) and, therefore, may be associated with a higher risk of development of new ischemic events.

### Some Issues with Regard to the Implication of HRV-Based Model for Ischemic Event Prediction

• *For patients with acute TIA*: Because the risk of secondary ischemic events after TIA is high in the first several hours and days (146, 147), the optimal design is to start ECG recording just after the occurrence of the TIA episode. Ideally, it would be best to recruit patients within 24 or 48 h of the TIA event and start recording ECG as soon as possible. The use of HRV can be compared with the traditional predictive ABCD2 score (or other tools such as ABCD3 and ABCD3-I), with regard to their predictive values on ischemic events after initial TIA or minor stroke.
• *Cutoffs of HRV for risk stratification*: Previous studies showed different cutoffs of HRV parameters, such as SDNN less than 50, or 70 or 100 ms, or HF less than 10 ms^2^, for the risk stratification of cardiovascular diseases (98, 148). Our view is that an attempt to find HRV cutoffs should be sought only in the context of a specific outcome and study population. Moreover, different HRV parameters may have different predictive values, which include both the absolute values and changes during a specific time period of each time domain, frequency domain, and non-linear parameter. Finally, the cutoffs depend on the sensitivity and specificity that clinicians/investigators select. Therefore, there may be no consensus on the ideal cutoffs for different HRV measures with regard to ischemic events occurrence. To define optimal cutoffs for different parameters or the best type of HRV parameter deserves further investigation.

## Summary

It is critical to estimate the risk of stroke occurrence or recurrence after initial TIA and to clearly identify those at a higher risk of developing secondary ischemic events among people with a burden of chronic risk factors/stressors. However, this remains challenging using current criteria, partly because the specific contributions of these risk factors are difficult to quantify given individual heterogeneity and also many other risk factors (unaddressed and unidentified) are not assessed when determining an individual’s risk profile. We have shown that both chronic risk factors and acute triggers of ischemic stroke are sources of stress to the body and are closely associated with ANS dysfunction that supports the neurogenic hypothesis of ischemic stroke development. The usual compensatory stress response of ANS may fail in the context of chronicity, which makes it challenged to face new acute stressors. The improper stress responses render these normally short-term responses prolonged and maladaptive, which relentlessly disrupts normal physiological pathways and progressively contributes to the development of stress-related diseases, such as TIA and ischemic stroke. Accordingly, we advocate that HRV assessment, as a measurement of ANS function, may represent the comprehensive effect of “multiple stressors” and may reflect the overall health condition at a given point in time. This review provides evidence for the use of HRV data to predict the occurrence of secondary ischemic events after initial TIA or minor stroke, as illustrated in Figure 3. This theoretical HRV-based comprehensive stress model and further hypotheses on identification of HRV predictors will initiate studies on identifying an innovative way to stratify the risk of TIA or minor stroke through assessing the effect of ANS and stress.

## Author Contributions

LG and J-PC proposed the study questions, conducted the literature review, and prepared the first draft of the manuscript. GM improved the study conception and provided a detailed review and editing. VC provided insight on specific aspects of the paper and provided a detailed review and editing. None of the authors received payment for writing the article. They all approve the submission of this version of the manuscript and take full responsibility for the manuscript.

## Conflict of Interest Statement

This publication is original: There are no prior publications or submissions with any overlapping information. None of the authors has disclosed any potential conflicts of interest. J-PC is supported in part by a scholarship of the BC Children’s Hospital Research Institute in Vancouver, Canada. The remaining authors have no financial relationships relevant to this article to disclose.
